# SONRISA: A Curriculum Toolbox for Promotores to Address Mental Health and Diabetes

**Published:** 2007-09-15

**Authors:** Kerstin M Reinschmidt, Jenny Chong

**Affiliations:** Canyon Ranch Center for Prevention and Health Promotion (previously known as Southwest Center for Community Health Promotion), Mel and Enid Zuckerman College of Public Health, University of Arizona; Native American Research and Training Center, Department of Family and Community Medicine, University of Arizona, Tucson, Arizona

## Abstract

**Introduction:**

SONRISA is a Spanish/English mental health curriculum toolbox developed for *promotores* (community health workers) who work with Hispanic clients to prevent or manage diabetes. *Promotoras* and community members from a community-based project requested their university partner to help *promotores* address depression observed in their clients with diabetes.

**Methods:**

Data collection included reviewing existing educational materials and conducting focus groups. *Promotoras* piloted the first version of SONRISA in a 1-day workshop.

**Results:**

Four curricula from community agencies were reviewed, and 49 individuals participated in eight focus groups. *Promotora* feedback during the workshop informed the revision of SONRISA.

The community-based participatory approach produced a highly relevant and culturally appropriate toolbox for general use by *promotores* and their clients. SONRISA provides training material to address depression and diabetes, educational material for clients, and approaches to prevent work-related emotional burnout.

**Conclusion:**

SONRISA offers an innovative, integrated approach to training *promotores* to address depression among their clients with chronic illnesses. It is culturally appropriate and adaptable to other populations.

## Introduction

Addressing comorbid mental health issues and chronic diseases simultaneously with the help of community health workers may increase the effectiveness of interventions aimed at chronic disease prevention and control. SONRISA (1) is a curriculum toolbox for *promotores* (community health workers [CHWs]) to use with their clients to address mental health issues (depression, stress, anxiety) and diabetes prevention and management. SONRISA is a community-based participatory project that makes use of the experience and expertise of community resources and personnel in every step of the project.

SONRISA was designed to address a gap in community health promotion materials for *promotores*. The aim was to design a multimodule curriculum toolbox that could be used to train *promotores* on how to address depression and diabetes at the patient, family, and community levels, and to offer educational material that can be integrated into existing diabetes curricula. We describe the collaborative efforts of university and community partners to develop the curriculum and explain how *promotores* implementing diabetes prevention or management programs can use it.

In Hispanic communities along the United States–Mexico border and throughout Mexico, CHWs are referred to as *promotores* or *promotoras* *de salud*, indicating CHWs or female CHWs, respectively. Throughout the article, *promotor(es)* is used as a general reference, whereas *promotoras* is used to refer specifically to the CHWs that worked with the SONRISA project.

SONRISA is not an acronym but a Spanish noun that directly relates to the positive outcomes of the program. It is derived from the Spanish verb *sonreir*. In English, the verb can be defined as "to convey (a feeling) with an expression," whereas, when used as an adjective, it can be defined as "having a bright or pleasing aspect." The English translation for the noun* sonrisa* is *smile*.

Hispanics, including Mexican Americans, suffer disproportionately from depression compared with other ethnic groups (2,3). Research findings point to the complex nature of this disparity.

Depression, stress, and psychological distress are closely linked with chronic diseases, including diabetes. Each increases the risk of developing the other, and each has negative effects on the other (2,4-11), including greater mortality (12) and greater and earlier incidence of adverse events (5). These events result in a substantially greater health burden among individuals with comorbid depression and diabetes than among those with diabetes only or among depressed individuals without diabetes (13).

Sex (2,3,14), socioeconomic status (2), culture (15-17), and acculturation (18) complicate the relationship between depression and chronic diseases, and may influence the prevalence and incidence of depression, thus contributing to the disparity in depression. The higher rates of depression among Hispanics (15) may be a function of a lower rate of insurance (19) and economic difficulties experienced by many minority groups. The impact of acculturation on depression is not as clear-cut (20-22) and may depend on other factors such as age (2,20) and sex (4,15,20,23,24).

Cultural factors may also contribute to the higher rate of depression among Hispanics compared with other U.S. groups. Stigma associated with depression leads Hispanics to be less likely to seek professional help (25). Hispanics are more likely than non-Hispanics to experience and express depression in somatic ways (e.g., stomachaches, headaches, dizziness) (15,26). When Hispanic patients choose not to discuss their true feelings (27), health care providers may mistake symptoms of depression for physical health issues or may misdiagnose the patients' condition altogether.

The literature on CHWs, including *promotores,* reflects their success in education (28), preventive health screenings (29,30), and chronic disease prevention and management interventions (31,32). Despite a gap in the research literature on the use and effectiveness of CHWs in mental health and chronic disease, CHWs based in clinical settings have found roles in both fields (33). As intermediaries between the community and local health care providers, CHWs are trusted community members who are an integral part of their community networks, striving to improve the health of their clients (34,35).

Designing and implementing CHW-based health programs is particularly important when working with sensitive issues, such as mental health, in ethnic minority communities. *Promotores* are uniquely qualified to address health disparities in culturally appropriate ways. As community members trained in chronic disease and mental health issues, they understand the nature of the local health care system and the availability of health-related resources. Their community membership and training help them take into account the different variables of chronic disease, age, sex, culture, and acculturation that play such crucial roles in the complex relationship between diabetes and depression. *Promotores* requested that their partners at the University of Arizona help to develop the curriculum toolbox to fill a void in educational materials that are useful in working with clients who have diabetes and are showing signs and symptoms of emotional distress.

## Methods

The SONRISA project was designed to ensure that the curriculum toolbox was culturally appropriate to the target population. Existing materials on depression and other mental health topics that were developed and used for the populations from border communities were reviewed. Several focus groups were conducted with individuals who were members of the target populations or who interacted with them. *Promotoras* evaluated the first version of the curriculum during a training workshop and provided constructive criticism and suggestions for the second version of the SONRISA toolbox. Data gathered for the SONRISA project during the focus groups and training/feedback workshop were examined for themes that were both identified in advance and newly emerging from the data. These themes informed the development and the revision of the curriculum toolbox. (A detailed analysis of the focus groups will be reported elsewhere.) The University of Arizona Institutional Review Board approved all parts of the project.

### Existing materials

Community health centers and community agencies serving Hispanic residents primarily in Santa Cruz and Yuma counties were contacted for educational material on mental health that they were using with their Hispanic clients with diabetes. During July and August 2004, materials from one community agency, one community program, and two community health centers were reviewed for design, content, and the presentation of information.

Campesinos sin Fronteras in Somerton shared educational material on depression, self-esteem, stress management, support groups, and family mental health (unpublished data, 2004). The developers of Compañeros en la Salud (36,37) shared their complete curriculum, which includes family mental health, stress management, family communication, and self-esteem. Platicamos Salud at the Mariposa Community Health Center in Nogales shared educational and presentation materials on depression, anxiety, and stress (unpublished data, 2004). The Western Arizona Health Education Center, Inc/Regional Center for Border Health (38) in San Luis shared a draft of its comprehensive mental health and substance abuse curriculum, H.E.R.O. (Helping Everyone Reach Out), which includes sections on self-esteem, stress control, depression, and anger.

All of the materials were bilingual (English and Spanish) and targeted at Hispanic communities. Except for H.E.R.O., which was aimed at *promotores* and not their clients, local materials presented information in multiple formats, including manuals for *promotores*, participant manuals, flipcharts, slide presentations, and protocols (including evaluation methods). The materials also shared certain characteristics in the way information was presented, including definitions, suggested exercises or activities, self-evaluations, checklists, visuals, motivational materials and stories, and quotes or sayings.

### Focus groups

In November and December 2004, and in March and May 2005, eight focus groups were conducted in four communities in Arizona close to the U.S.–Mexico border. Six of the focus groups were conducted with groups in Cochise County: one each with community service providers, patients, family members of patients, *promotoras*, and two groups of community members. Providers and *promotoras* had been asked to assist the study as focus group participants and to help recruit patients and their family members for the focus groups. Community members were recruited into focus groups at the suggestion of local community policy group members and on the basis of word-of-mouth referrals by Cochise County *promotoras*. At the request of *promotoras* working in Yuma and Santa Cruz counties, two more focus groups were added.

A total of 49 individuals participated in the study. Providers from a community health center in Cochise County helped recruit health care providers (n = 11). Cochise County community members suggested community focus group participants (n = 4 and n = 5), and *promotoras* in Cochise County recruited patients (n = 3) and these patients' family members (n = 6). *Promotoras* from Yuma County (n = 11) participated in focus groups, as did *promotoras* from Santa Cruz County (n = 7) and Cochise County (n = 2).

Except for one focus group that took place at a community health center, all focus groups were held in public locations. The focus groups lasted from 45 to 150 minutes.

During the focus groups, participants were asked about their attitudes and experiences with depression among individuals with diabetes and their recommendations about how to identify people with diabetes who are depressed and support them at work or at home. All focus group discussions were structured with eight questions and probes for each question, according to focus group question guidelines. Each guideline asked for the information described above but was adapted to the particular focus group population. During the focus groups, all participants shared their thoughts and experiences. More reserved participants were encouraged to speak. Focus groups were tape-recorded and transcribed to ensure accurate documentation of the proceedings. All participants signed a consent form before participating in the focus group.

### Pilot test

The developers of the curriculum toolbox participated in the training workshop; the main developer (the first author) facilitated the training. *Promotoras* were recruited through the Arizona Community Health Outreach Workers Network, Inc. Workshop participants were introduced to the newly developed curriculum material and asked to answer questions in the Post Training Evaluation Question Guide. The questions inquired about the usefulness, completeness, and cultural appropriateness of the educational material. The proceedings were recorded manually, tape-recorded, and transcribed. All participants signed a consent form before participating in the pilot test.

## Results

The focus groups, the pilot test, and personal communication with community partners who shared their materials provided information directly relevant to the curriculum design, contents, and format. Thirteen *promotoras* participated in the 1-day training/feedback workshop in May 2006. Results of the workshop posttraining evaluation revealed that participants found the first version of SONRISA to be a useful curriculum toolbox. They considered the information on mental health issues to be comprehensive and useful for their work in diabetes prevention and management with their patients, their patients' families and friends, and the communities. The *promotoras'* suggestions for improvement included 1) changing some of the words to everyday language because English was the second language for many *promotores;* 2) having dividers and adding more headings, stories, case studies, role play, and color; and 3) combining sections.

Participants suggested that five types of information be included in the curriculum:

1. Basic information on depression, its signs and symptoms, severity levels (i.e., mild, moderate, and severe), where to send clients for help, and treatment.

2. Culturally recognizable signs and symptoms of depression (including anger or irritation, the tendency to quarrel, and changes in personality), cultural explanations of depression (such as *susto* or *nervios*), and a reference to the tendency of Mexican Americans to stigmatize depression and to consider someone with a diagnosis of depression as *loco/loca* (i.e., crazy).

3. Indicators of stress and anxiety, because both go hand in hand with depression.

4. Specific needs of persons with diabetes, their families, and community members, such as knowing how diabetes affects a person physically and mentally, how to control blood glucose levels, how to make family members understand what is happening with the person who has diabetes, and how to get free or affordable health care services.

5. Specific needs of *promotores* working with individuals and groups on diabetes/depression prevention and management, including knowing how to identify (but not diagnose) depression, preferably with the help of a screening tool; how to communicate with clients to avoid offending or stigmatizing them; how to help clients; when and where to refer clients; and coping skills for *promotores* for their own stress to prevent work-related emotional burnout.

It was determined that the curriculum should be written in English and in Spanish, be user-friendly, and present scientific information in language that is easy to understand. Examples were encouraged. SONRISA should offer modules that could be used for individual, family, and community interventions. Although the curriculum should have a structure, it should also allow for flexibility and allow *promotores* to select which modules and information to use. Importantly, the curriculum toolbox should take a relational approach (i.e., it should focus on positive aspects of social relationships, using social events such as sports that would allow depression to be addressed indirectly and in social context [FG Castro, PhD, Arizona State University, oral communication, July 2004]). A higher quality of interpersonal functioning (i.e., social support) has been found to protect against depression, although the process has not been well described and requires further study (39).

The first version of SONRISA contained 10 major sections addressing the issues identified from the existing materials and the focus groups. This version of SONRISA was revised on the basis of feedback from the training workshop. The previous 10 sections were reduced to six main sections plus acknowledgments, a note on visuals, appendices, and references. At the beginning of each section, a few paragraphs summarize the section content and offer suggestions on use. The presentation of the materials was improved by added dividers and more headings. Visual markers (i.e., icons) now orient the curriculum toolbox user to the origins (e.g., summary of focus group statements) or intended uses of specific materials (e.g., handout, note to *promotores*, suggestion for group discussion or group activity). Pages not marked by visual markers are intended as background material for the *promotores*. Clip art adds color to the pages and emphasizes the topic under discussion.

To validate that the information given is accurate, reports from scientific journals were also included. Materials adapted from materials contributed by community partners are referenced throughout the curriculum. Short quotes from project participants were added, as were suggestions for discussion topics and group activities. An attempt was made to simplify the language throughout the curriculum. A bilingual native Spanish speaker translated the second version of the English curriculum into Spanish.

The sections of the second version of SONRISA are shown in the abbreviated bilingual table of contents (Figure 1). Most of the sections that make up SONRISA simultaneously inform about mental health issues and function as educational material for workshops or classes either for *promotores* or their clients. Curriculum sections and subsections are accompanied by guidelines and suggestions on how to use the subsequent materials. Sections 1, 2, 5, and 6 are intended for use by the *promotores*. Information presented in sections 3 and 4 and the appendices can be used by *promotores* as teaching materials for their clients, or for themselves — individually, during trainings, or in support groups. Section contents are summarized in Figure 2.

**Figure 1.** *SONRISA*, Table of Contents.

SONRISA Table of Contents	SONRISA Índice de Contenido
Acknowledgments	Agradecimientos
A note on visuals	Una nota sobre los símbolos visuales
1. Introduction	1. Introducción
2. The roles of *promotores*/community health workers (CHWs) in helping clients to prevent and manage mental/emotional health issues related to diabetes	2. Funciones de los promotores/ trabajadores de salud comunitaria (TSC) en ayudar a prevenir o manejar la mala salud mental/emocional relacionada con la diabetes
3. Basics of mental/emotional health issues 3.1. The importance of addressing issues of mental/emotional health 3.2. Depression 3.3. Stress 3.4. Anxiety	3. Lo fundamental de los problemas mentales/emocionales 3.1. La importancia de resolver asuntos de la salud mental/emocional 3.2. Depresión 3. Estrés. 4. Ansiedad
4. *Promotores*/community health workers (CHWs) helping clients to cope 4.1. Working with patients 4.2. Working with patients, family members, and friends 4.3. Working with community members	4. Promotores/trabajadores de salud comunitaria (TSC) ayudando a los clientes a enfrentar la diabetes 4.1. Trabajando con los pacientes 4.2. Trabajando con pacientes, familiares, y amigos 4.3. Trabajando con los miembros de la comunidad
5. Coping strategies and priority setting for *promotores*/community health workers (CHWs)	5. Mecanismos de adaptación y establecimiento de prioridades para promotores/trabjadores de salud comunitaria
6. Resources for *promotores*/CHW trainings 6.1. Evaluation questions 6.2. SONRISA training certificate: Celebrating our success	6. Recursos para entrenamientos de *promotores*/TSC 6.1. Preguntas de evaluación 6.2. Certificado de entrenamiento SONRISA: Celebrando nuestro éxito
Appendices	Apendices
References	Referencias

**Figure 2.** SONRISA, Section Contents.

Section	Content
1	Introduction to SONRISA and why and how it was developed.
2	Discussion of the roles of *promotores* in helping to prevent or manage mental health issues related to diabetes.
3	Basic information on depression, stress, and anxiety.
4	Suggestions for working with patients, families, or community members in preventing and managing depression, stress, and anxiety associated with diabetes.
5	Information that is targeted at helping *promotores* avoid or manage occupational burnout by improving coping skills and setting priorities.
6	Administrative material that invites *promotores* to participate in improving this toolbox, and suggestions for a certificate template should SONRISA material be used for training others.
Appendices	Additional information, resources, and teaching materials.

## Discussion

The high prevalence and complex nature of depression in Hispanic populations call for innovative, community-based, participatory approaches for the prevention and early detection of depression, especially among those suffering from chronic diseases. *Promotores* working with clients with diabetes along the U.S.–Mexico border near Arizona, as well as community members interested in community health, identified the need for resources that *promotores* could use to address their clients' mental health needs while working with them on diabetes management or prevention.

Until now, few appropriate educational materials on mental health have been available for Hispanic residents with diabetes along the U.S.–Mexico border near Arizona. SONRISA is an important and timely toolbox for *promotores*. Through the collaborative efforts of border communities with the University of Arizona, a curriculum toolbox was developed on the basis of a community-identified need. The curriculum toolbox is culturally appropriate for the Hispanic population, was informed by stakeholder focus group participants, and was further improved through feedback from the people for whom it was designed. Comments directly relevant to the curriculum design, contents, and format came from focus groups and from personal communication with community partners who shared their materials.

SONRISA is a curriculum toolbox in the sense that its tools can be used individually or in combination with other tools to produce a more comprehensive and relevant diabetes curriculum. It has been made available to other organizations and *promotores*, and other workshops have been held.

Because the SONRISA curriculum project was developed with *promotores* from the U.S.–Mexico border near Arizona, the number of focus group participants was small. Future work should evaluate the use and effectiveness of the curriculum materials when used in conjunction with diabetes intervention programs in other border communities.

We expect that the culturally appropriate approaches embodied by *promotores* along the U.S.–Mexico border near Arizona and the long overdue intervention approach that integrates chronic disease and mental or emotional health in public health intervention materials will significantly affect the prevention and management of diabetes and associated depression on Hispanic border populations in Arizona. Studies testing this impact should be implemented.

The challenges and lessons learned from this project can be useful to others who plan to adapt this curriculum or who plan to design their own. The main challenges encountered during the SONRISA project were logistical: the small number of focus group participants and difficulty in scheduling the focus groups. Because border communities have only small populations to recruit from, the researchers should have invited focus group participants from more than one border community right from the start of the project. Broadening the study population would be within the spirit of the project, namely to design a curriculum toolbox for the border population. The *promotoras* in Yuma and Santa Cruz counties deserve credit for their self-recruitment into the project. The difficulty in scheduling focus groups at a time convenient for participants and researcher teams alike could have been addressed by allowing more time for the project.

SONRISA is sufficiently flexible to be adapted for other populations and other chronic disease interventions and curricula. The curriculum provides the framework within which material pertinent to other cultures (e.g., signs of depression, suggestions for culturally appropriate activities) can be inserted. It can also be modified for other Hispanic populations and revised on the basis of further trainings, research, and emerging partnerships with other community agencies. Since SONRISA was conceptualized as a "living toolbox," we hope that the SONRISA project will continue to be enhanced as community organizations obtain the toolbox, use its materials, and further improve it.
